# Alleviation of tissue adhesion using dual-functional methacrylated gelatin via immunomodulation and antifibrotic activity

**DOI:** 10.3389/fimmu.2026.1777773

**Published:** 2026-03-04

**Authors:** Pei Yuan, Shichun Feng, Chong Tang, Xuesong Gao, Shengkui Qiu, Geshuyi Chen

**Affiliations:** 1 Gastrointestinal Surgery Department, Nantong First People’s Hospital, Southeast University, Nantong, Jiangsu, China; 2 Gynecology Department, Nantong First People’s Hospital, Southeast University, Nantong, Jiangsu, China

**Keywords:** biomaterials, houttuynia cordata extract, hydrogel, intestinal adhesion, nintedanib, tissue engineering

## Abstract

Intestinal adhesion is one of the most prevalent clinical conditions, affecting millions of patients globally. However, effective strategies to decrease the incidence of intestinal adhesion remain insufficient. In this study, we developed a novel and unreported dual-functional methacrylated gelatin (nhGelMA) that can regulate inflammation and antifibrotic functions by integrating gelatin methacryloyl (GelMA), Houttuynia cordata extract, and nintedanib. First, optimal concentrations of GelMA hydrogels were prepared and screened, followed by the selection of appropriate concentrations of Houttuynia cordata extract and nintedanib to prepare the final nhGelMA. Subsequently, nhGelMA was characterized, and its biocompatibility and biological functions were analyzed. The results indicated that nhGelMA exhibited good biocompatibility, no organ toxicity, and the ability to modulate the expression level of inflammation and fibronectin (FN). Finally, nhGelMA hydrogel was applied in the preventive treatment of hemorrhagic intestinal adhesion, and the results indicated that nhGelMA effectively reduced the deposition of FN, laminin (LAMA), and collagen type I (Col1), as well as decreased the infiltration of macrophages and neutrophils, thereby preventing the occurrence of intestinal adhesion. Importantly, the further transcriptomics demonstrated that nhGelMA could regulate protein digestion, absorption, and adhesion, as well as reconstruct the anatomical structure, contributing to the alleviation of hemorrhagic intestinal adhesion.

## 1 Introduction

Intestinal adhesion is a prevalent clinical condition, which affects millions of patients globally (1–5). In general, the incidence rate of intestinal adhesion exceeds 50% after injuries or surgeries involving the abdominal cavity (2, 4, 6). Patients with intestinal adhesion experience intermittent abdominal colic, which can progress to mechanical intestinal obstruction, necessitating surgical intervention during episodes of intestinal dysfunction (7–9). Therefore, preventing the occurrence of intestinal adhesion is critical for reducing postoperative adverse symptoms and enhancing the quality of life of patients (2, 4, 8, 10, 11). However, effective strategies to decrease the incidence of intestinal adhesion remain insufficient.

Tissue engineering and biomaterials present promising approaches for mitigating intestinal adhesion (12–16). Based on previous studies, abnormal tissue healing caused by bleeding and inflammation in the abdominal cavity primarily contributes to intestinal adhesion (17). Furthermore, the secretion and deposition of fibronectin serve as key linkers in intestinal adhesion (17). Therefore, regulating the secretion and deposition of fibronectin is crucial for preventing the occurrence of intestinal adhesion. Meanwhile, fibronectin is primarily synthesized and secreted by fibroblasts and macrophages (18). Thus, a dual-dimensional approach that targets the cellular level (by reducing the infiltration of inflammatory cells and fibroblasts) and the molecular level (by decreasing the synthesis and secretion of fibronectin) is a promising and effective strategy to alleviate intestinal adhesion.

Houttuynia cordata extract, a traditional herbal component, exhibits excellent biocompatibility, anti-inflammatory, low cost, and high potential for clinical translation properties (19, 20). Nintedanib, which is an antifibrotic agent with hypotoxicity, could effectively inhibit the generation and deposition of fibronectin (21, 22). Therefore, integrating Houttuynia cordata extract with nintedanib can alleviate the factors influencing intestinal adhesion at cellular and molecular levels. Furthermore, the early phase of acute inflammation and hemorrhagic exudation is a critical period that promotes the accumulation of factors contributing to intestinal adhesion, during which the continuous regulation of local inflammatory factors influencing intestinal adhesion is necessary (23). However, the combination of Houttuynia cordata extract and nintedanib alone may not suffice the demand for continuous treatment. GelMA, which is a popular hydrogel, has good biocompatibility and plasticity, making it suitable for applications in tissue regeneration and repair (24, 25). Its loosely crosslinked network enables sustained drug release, thereby addressing the need for continuous and stable drug delivery (25, 26). Moreover, the integration of GelMA, Houttuynia cordata extract, and nintedanib to alleviate hemorrhagic intestinal adhesion has not been reported to date.

**Figure 1:**
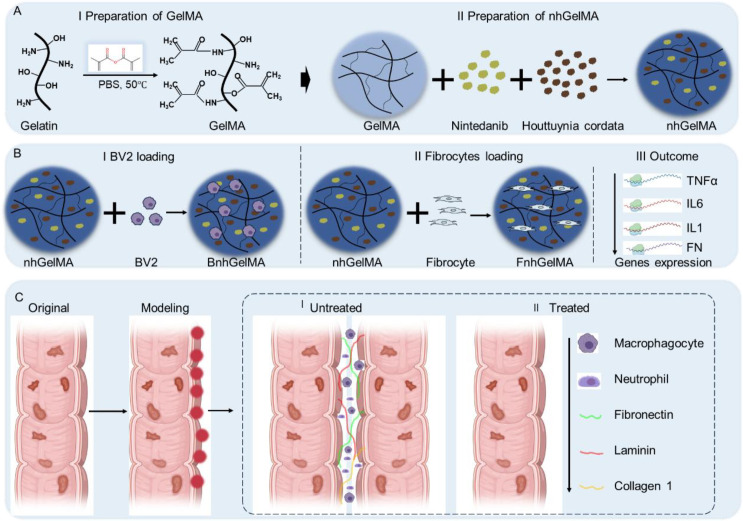
Schematic illustration of alleviation hemorrhagic intestinal adhesion using dal-functional methacrylated gelatin (nhGelMA) via immunomodulation and anti-fibrosis. **(A)** The preparation process of nhGelMA is detailed. **(B)** The evaluation of nhGelMA functions pertaining to immunomodulation and anti-fibrosis *in vitro*. **(C)** The application of nhGelMA *in vivo* demonstrates its efficacy in alleviating hemorrhagic intestinal adhesion.

Thus, in this study, GelMA, Houttuynia cordata extract, and nintedanib were integrated to develop a dual-functional methacrylated gelatin (nhGelMA) that can regulate inflammation and antifibrotic functions (**Figures 1A–C**). First, optimal concentrations of GelMA hydrogels were prepared and screened, followed by the selection of appropriate concentrations of Houttuynia cordata extract and nintedanib to prepare the final nhGelMA. Subsequently, nhGelMA was characterized, and its biocompatibility and biological functions were analyzed. The results indicated that nhGelMA exhibited good biocompatibility, no organ toxicity, and the ability to modulate the expression level of inflammation and fibronectin (FN). Finally, nhGelMA hydrogel was applied in the preventive treatment of hemorrhagic intestinal adhesion, and the results indicated that nhGelMA effectively reduced the deposition of FN, laminin (LAMA), and collagen type I (Col1), as well as decreased the infiltration of macrophages and neutrophils, thereby preventing the occurrence of intestinal adhesion. Importantly, the further transcriptomics demonstrated that nhGelMA could regulate protein digestion, absorption, and adhesion, as well as reconstruct the anatomical structure, contributing to the alleviation of hemorrhagic intestinal adhesion.

## 2 Results and discussion

### 2.1 Preparation of nhGelMA

Previous studies have indicated that abnormal tissue repair resulting from injury, bleeding, and inflammatory infections is the primary factor contributing to intestinal adhesion (12–14). Furthermore, the secretion and deposition of fibronectin serve as critical linkers in the formation of intestinal adhesion, which are primarily synthesized and secreted by fibroblasts and macrophages (18). Therefore, using a dual-dimensional approach aimed at reducing the infiltration of inflammatory cells and fibroblasts, as well as the synthesis and secretion of fibronectin, has great application potential in developing an effective repair strategy to alleviate intestinal adhesion. The development of hydrogels and drugs provides the technical support necessary to meet the aforementioned requirements for mitigating intestinal adhesion.

**Figure 2:**
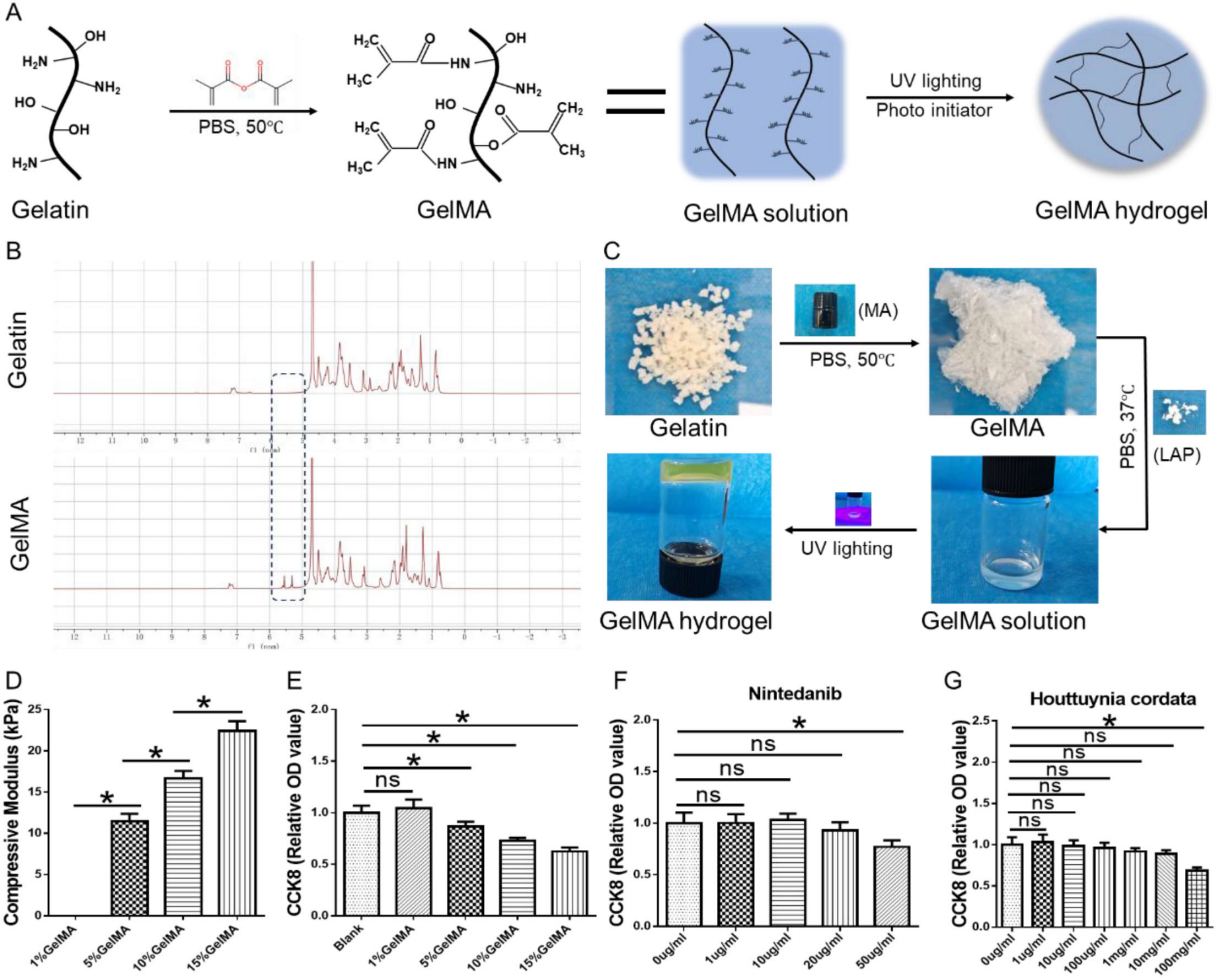
Preparation of nhGelMA. **(A)** A schematic illustration of the synthesis process for nhGelMA. **(B)** The NMR-H spectra of gelatin and GelMA. **(C)** Gross views of the synthesized nhGelMA. **(D)** The compressive modulus of 1%, 5%, 10%, and 15% GelMA. **(E)** The CCK8 test results for the Blank, 1% GelMA, 5% GelMA, 10% GelMA, and 15% GelMA groups. **(F)** CCK8 test results for nGelMA with 0, 1, 10, 20, and 50 µg/ml of nintedanib. **(G)** CCK8 of hGelMA with 0, 1, 10, 100, 1000,10000, and 100000 ug/ml of houttuynia cordata extract. *P < 0.05. ns *P* > 0.05.

**Figure 3:**
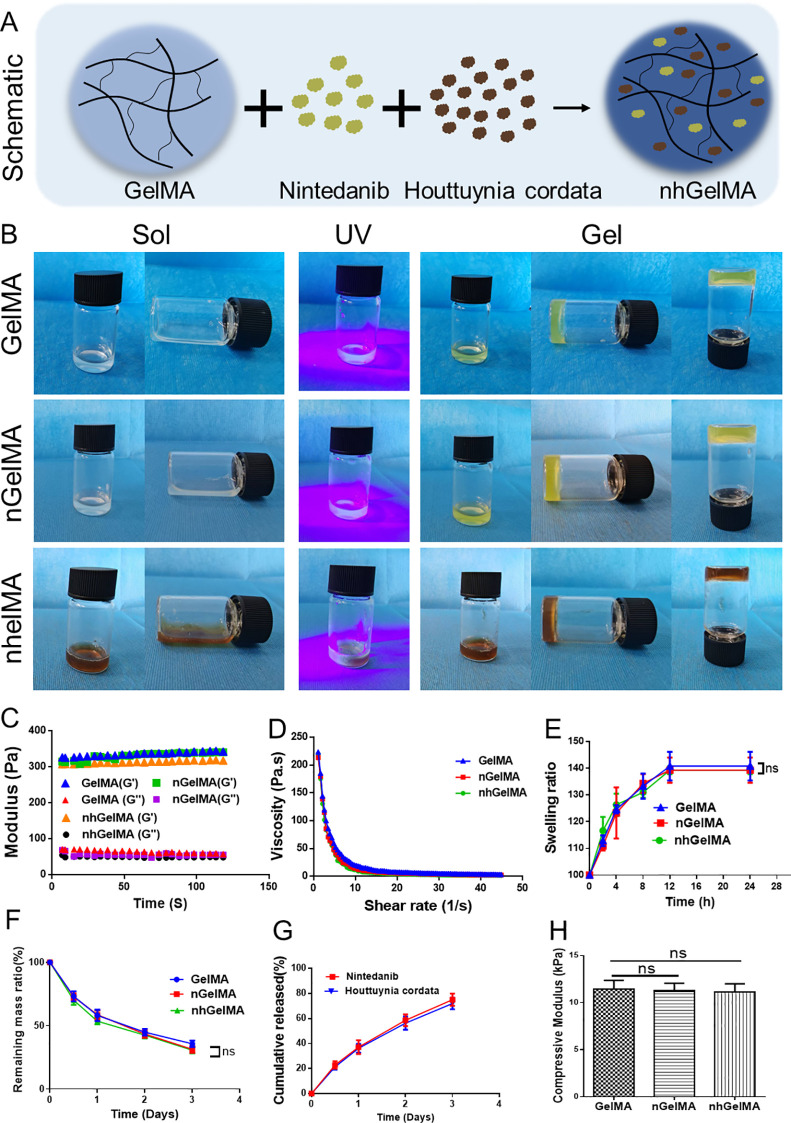
Characterizations of the hydrogels. **(A)** The schematic illustration of nhGelMA preparation. **(B)** The gross views of GelMA, nGelMA, and nhGelMA hydrogels before and after UV-irradiation. **(C–F)** Rheological analyses **(C)**, viscosity **(D)**, swelling ratio **(E)**, and degradation **(F)** in GelMA, nGelMA, and nhGelMA groups. **(G)** The cumulative release of nintedanib and houttuynia cordata extract. **(H)** The compressive modulus for GelMA, nGelMA, and nhGelMA hyrdogels. ns *P* > 0.05.

Gelatin, which is a natural material, has been widely applied in daily life and clinic because of its excellent biocompatibility and biosafety. The exposed amino (–NH2) and hydroxyl (–OH) groups on the amino acid chains of gelatin provide outstanding plasticity and serve as a chemical basis for the modification required to prepare methacryloylated gelatin (GelMA) (27, 28). The carbon–carbon double bonds of GelMA could be activated under the simultaneous excitation of the photoinitiator (LAP) and ultraviolet light, forming new links that lead to a network structure, which macroscopically manifests as a hydrogel (**Figure 2A**). The convenience of the gelation of GelMA hydrogels, along with the crosslinked network formed after gelation, provides an operational and structural basis for drug loading and release, making it an excellent drug carrier. In this study, GelMA was synthesized, and nuclear magnetic resonance hydrogen spectroscopy (NHR-H) was performed on the gelatin and GelMA groups to confirm chemical grafting. The results indicated that the GelMA group exhibited double peaks corresponding to successful grafting between 5 and 6 ppm (**Figure 2B**). Furthermore, the gross views of GelMA preparation and hydrogel formation are presented in **Figure 2C** to confirm the successful preparation of the hydrogel. The results indicated that GelMA was successfully synthesized, and hydrogels were formed. The concentration of the hydrogel remarkably influences the density of the crosslinked network within the scaffold, thereby affecting its mechanical strength and nutrient permeability. At a hydrogel concentration of 1%, the internal crosslinked network fails to form sufficient mechanical strength to maintain its macroscopic bulk morphology, thereby resulting in a fluid state that is unsuitable for subsequent drug loading. As the concentration increases, the mechanical strength of the hydrogel escalates (**Figure 2D**). However, enhancing strength to encapsulate cells leads to a reduction in cell viability with increasing concentration (**Figure 2E**). Therefore, considering the operational feasibility and the impact on cellular viability, 5% GelMA concentration was used for the subsequent experiments. In this study, Houttuynia cordata extract, which is a traditional natural herbal component, showed excellent biocompatibility and anti-inflammatory functions, which were used to modulate inflammatory cells following intestinal injury. However, the optimal dosage remains to be confirmed. Consequently, GelMA was prepared using varying concentrations of Houttuynia cordata extract (hGelMA) to assess its toxicity on fibroblasts. The results indicated that Houttuynia cordata extract is safe at concentrations below 10 mg/mL (**Figure 2G**). Therefore, 10 mg/mL Houttuynia cordata extract was selected for subsequent experiments. Similarly, nintedanib, which is a well-established antifibrotic drug, was utilized to diminish FN production in this study. The cytotoxicity tests conducted at various concentrations revealed that nintedanib is safe at concentrations below 20 µg/mL (**Figure 2F**). Thus, 20 µg/mL nintedanib was established for subsequent experiments. Finally, nhGelMA hydrogel was successfully prepared by mixing 20 µg/mL nintedanib and 10 mg/mL Houttuynia cordata extract into 5% GelMA (**Figure 3A**).

### 2.2 Characterizations of the hydrogels

**Figure 4:**
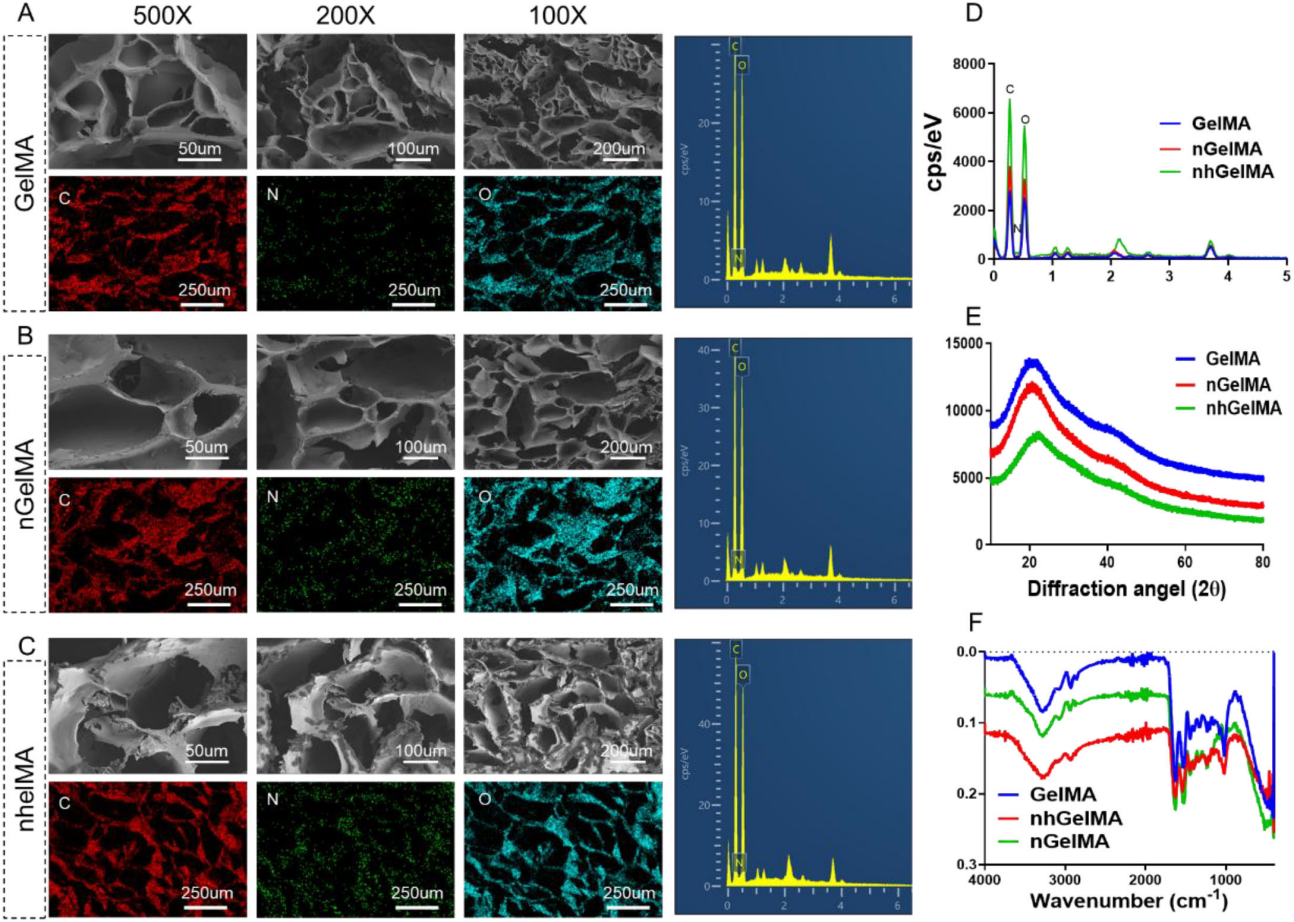
The structure and element of the hydrogels. **(A-C)** The SEM and elemental distribution images in GelMA, nGelMA, and nhGelMA groups. **(D)** The quantitative analysis of carbon, nitrogen, and oxygen. **(E, F)** The XRD and FTIR of GelMA, nGelMA, and nhGelMA.

Further verification of the influence of the incorporated Houttuynia cordata extract and nintedanib on hydrogel characterization is necessary to evaluate their safety and feasibility. The gross views of the transition from sol to gel for GelMA, hGelMA, and nhGelMA, along with rheological and viscosity assessments, indicated that the incorporation of Houttuynia cordata extract and nintedanib does not affect the rheological properties (**Figures 3B–D**). The results of swelling tests indicated that the swelling ratio of the hydrogels is below 150%, which is considered as a safe range for swelling in the peritoneal cavity (**Figure 3E**). Furthermore, degradation experiments revealed that GelMA, hGelMA, and nhGelMA exhibited similar degradation rates, degrading over 50% within 2 days, thereby confirming their biodegradability and safety (**Figure 3F**). The sustained release of Houttuynia cordata extract and nintedanib in nhGelMA is an important characteristic for the current repair strategy. The *in vitro* sustained release results confirmed that Houttuynia cordata extract and nintedanib could release over 60% of the drug within 3 days (**Figure 3G**), indicating the application potential of nhGelMA in achieving a time-matched release to regulate inflammatory cells and reduce fibronectin generation after intestinal damage. Moreover, the compressive modulus of GelMA, hGelMA, and nhGelMA indicated that the addition of Houttuynia cordata extract and nintedanib does not affect the mechanical properties (**Figure 3H**). In addition, SEM analysis showed that the hydrogels possessed similar porous network structures (**Figures 4A–C**). Elemental analysis revealed that the incorporation of Houttuynia cordata extract and nintedanib increased the ratio of carbon (C) and oxygen (O) (**Figures 4A–D**). Furthermore, the results of XRD and FTIR indicated similar crystalline structures and chemical bonds in GelMA, hGelMA, and nhGelMA hydrogels (**Figures 4E, F**).

### 2.3 Biocompatibility of the hydrogels

**Figure 5:**
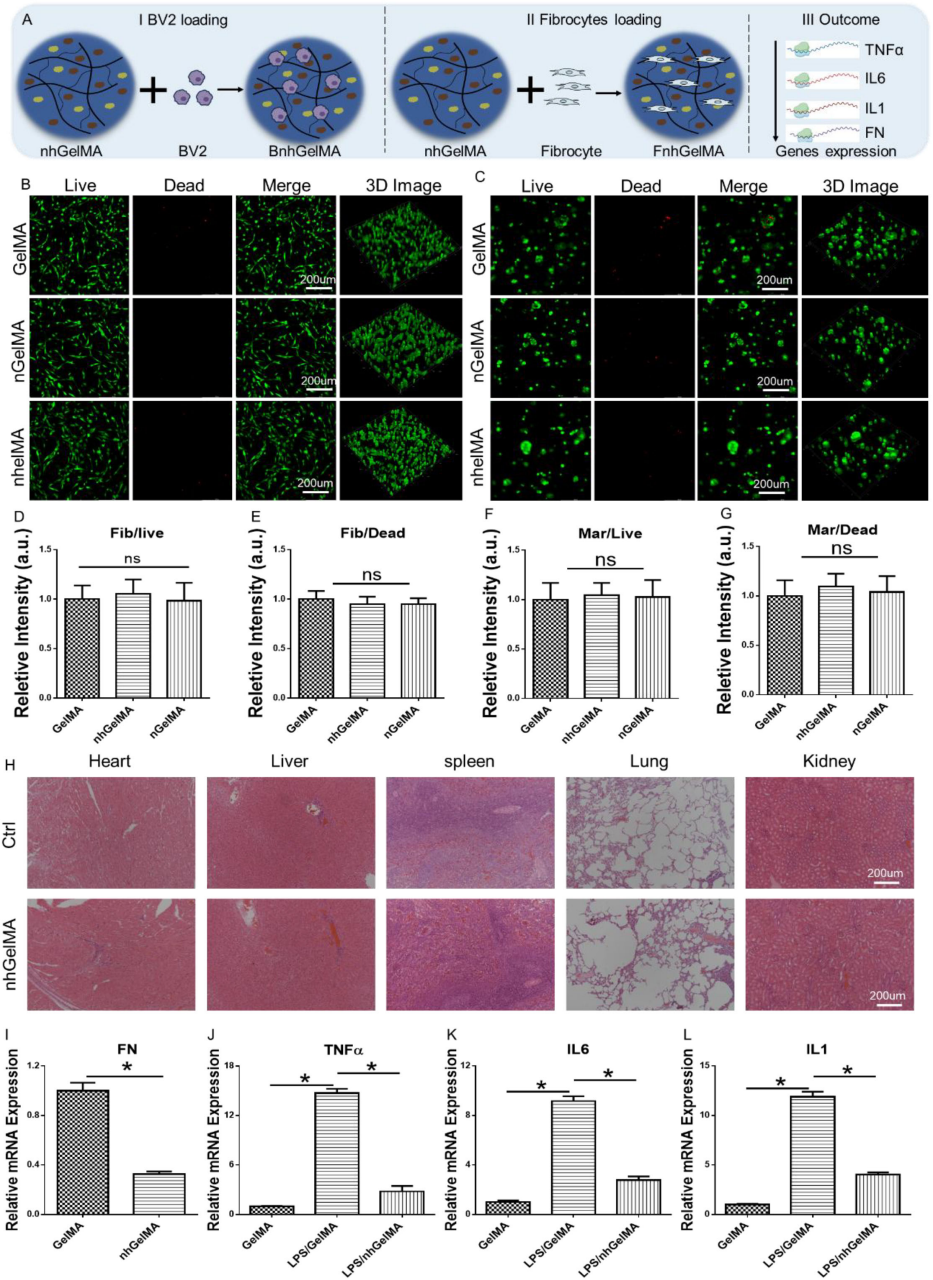
Biocompatibility and organ toxicity. **(A)** The schematic illustration of evaluating nhGelMA functions pertaining to immunomodulation and anti-fibrosis *in vitro*. **(B, C)** The live/dead staining of fibrocytes and BV2 loaded GelMA, nGelMA, and nhGelMA hydrogels at 3 days. **(D-G)** The quantitative analysis of live/dead staining for fibrocytes and BV2. **(H)** Organ toxicity of nhGelMA. **(I-L)** The genes expressive level of *FN* **(I)**, *TNFα* **(J)**, *IL6* **(K)**, and *IL1* **(L)**. *P < 0.05. ns *P* > 0.05.

The biocompatibility of nhGelMA is crucial for its potential *in vivo* applications, which needs further clarification. In this study, fibroblasts (the primary cells responsible for FN production) and macrophages (the main cells that secrete inflammatory factors) were selected as validating seed cells to evaluate its biocompatibility (**Figure 5A**). The results of live/dead staining indicated that fibroblasts and macrophages survived well in GelMA, hGelMA, and nhGelMA with only a few dead cells (**Figures 5B–G**). In addition, assessing whether the application of the hydrogel *in vivo* causes organ toxicity is important. The results of H&E staining indicated that the application of nhGelMA *in vivo* did not lead to significant changes in the heart, liver, spleen, lungs, or kidneys compared with an untreated control group, thereby confirming the absence of organ toxicity (**Figure 5H**). Finally, gene expression analyses related to FN and inflammatory factors were conducted in the GelMA and nhGelMA groups to further investigate the functions of Houttuynia cordata extract and nintedanib in nhGelMA *in vitro*. The results indicated that nhGelMA could reduce the expression level of the *FN* gene in fibroblasts, as well as the expression level of the inflammatory genes *TNFα*, *IL6*, and *IL1* in macrophages (**Figures 5I–L**), implying the excellent anti-inflammatory properties of nhGelMA and Houttuynia cordata extract. Therefore, nhGelMA has predefined antifibrotic and inflammatory regulatory functions, making it suitable for further *in vivo* experiments aimed at alleviating intestinal adhesion.

### 2.4 The application of nhGelMA in alleviating hemorrhagic intestinal adhesion

**Figure 6:**
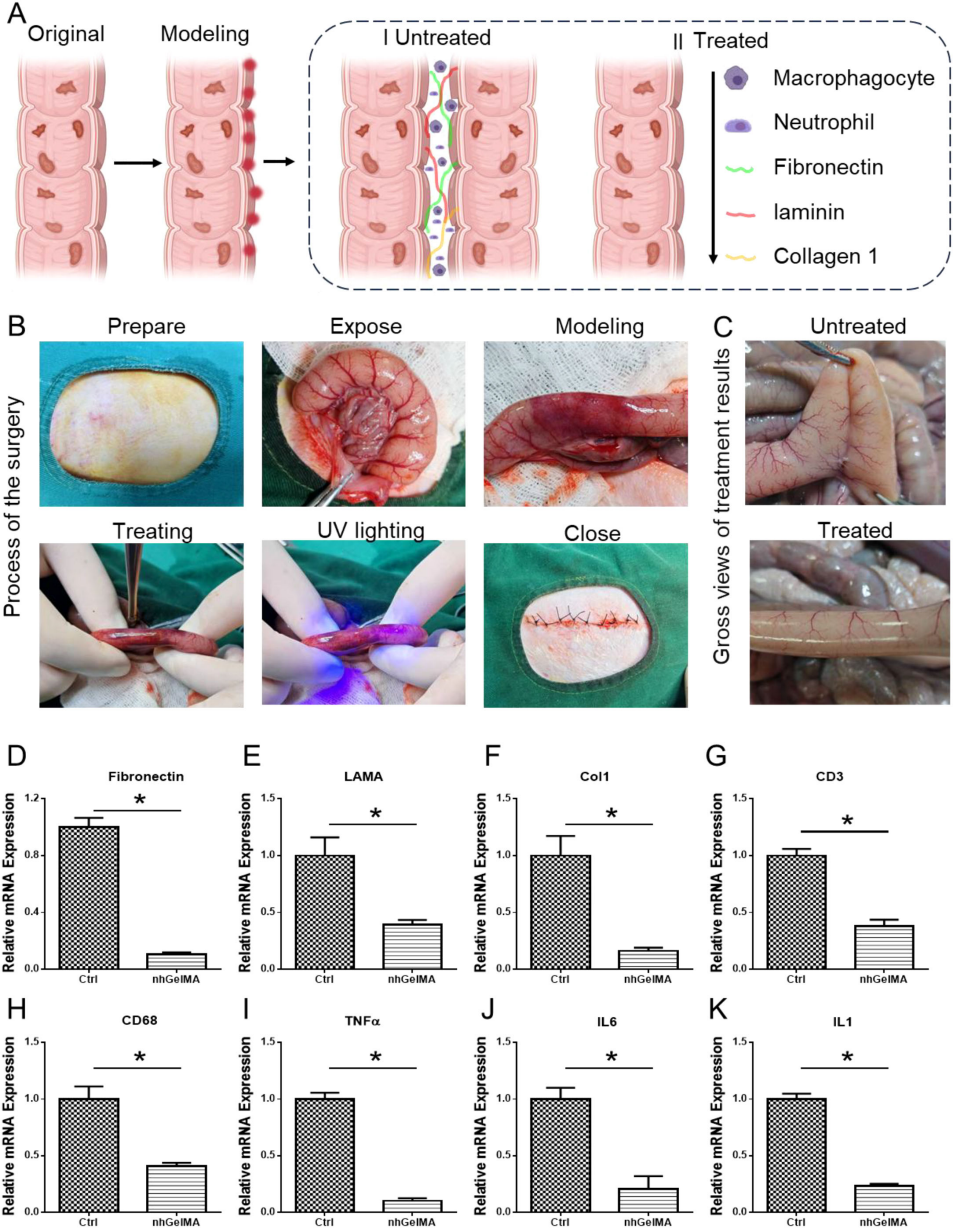
The application of nhGelMA in alleviating hemorrhagic intestinal adhesion. **(A)** The schematic illustration of the process and mechnism in alleviating hemorrhagic intestinal adhesion. **(B)** The process of the surgery. **(C)** The gross views of the untreated and treated intestine. **(D-K)** The genes expressive level of *FN* **(D)**, *LAMA* **(E)**, *Col1* **(F)**, *CD3* **(G)**, *CD68* **(H)**, *TNFα* **(I)**, *IL6* **(J)**, and *IL1* **(K)**. *P < 0.05.

**Figure 7:**
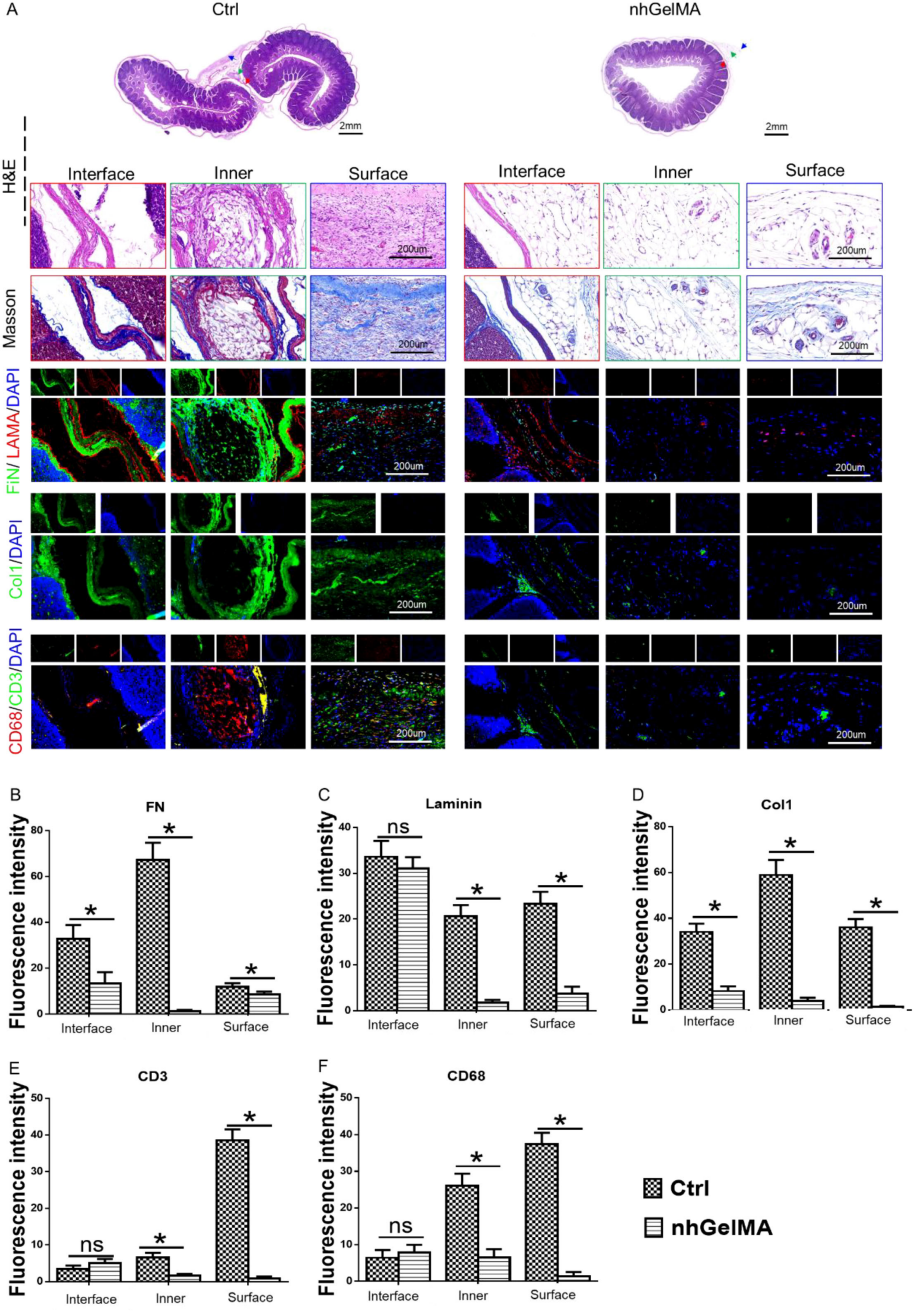
Histological staining of untreated and treated intestine. **(A)** The H&E, Masson, FN/LAMA/DAPI, Col1/DAPI, and CD68/CD3/DAPI staining in Ctrl and nhGelMA groups. **(B-F)** The quantitative analysis of fluorescence intensity in FN **(B)**, LAMA **(C)**, Col1 **(D)**, CD3 **(E)** and CD68 **(F)**. *P < 0.05. ns *P* > 0.05.

The ability of nhGelMA to effectively alleviate intestinal adhesion is the most important aspect in this study. The untreated intestinal injury served as the control group, whereas the experimental group received nhGelMA treatment (**Figure 6B**). The gross views made 3 days postsurgery indicated that the untreated group developed severe intestinal adhesion, whereas the nhGelMA group exhibited no adhesion (**Figure 6C**). Furthermore, PCR analysis was performed in the control and nhGelMA groups to further elucidate the mechanism by which nhGelMA alleviates adhesion. The results indicated that nhGelMA not only reduced the expression level of adhesion-related genes, namely, *FN* (**Figure 6D**), *LAMA* (**Figure 6E**), and *Coll* (**Figure 6F**), but also downregulated the expression level of inflammation-related genes, namely, *CD3* (**Figure 6G**), *CD68* (**Figure 6H**), *TNFα* (**Figure 6I**), *IL6* (**Figure 6J**), and *IL1* (**Figure 6K**). Further histological staining with H&E, Masson, FN, LAMA, and Col1 in the untreated and nhGelMA groups revealed the remarkable accumulation of adhesion-related proteins in the adherent intestinal wall of the untreated group (**Figure 7**). By contrast, only a small amount of these proteins was found in the treatment group, indicating that nhGelMA hydrogel plays a crucial role in reducing adhesion (**Figures 7A–D**). In addition, inflammation-related staining for CD68 and CD3 showed that the inflammatory infiltration in the untreated group was significantly higher than that in the control group (**Figures 7A, E, F**). Therefore, nhGelMA can alleviate intestinal adhesion by reducing adhesion and inflammation (**Figure 6A**).

### 2.5 The mechanism analysis of nhGelMA in alleviating hemorrhagic intestinal adhesion

**Figure 8:**
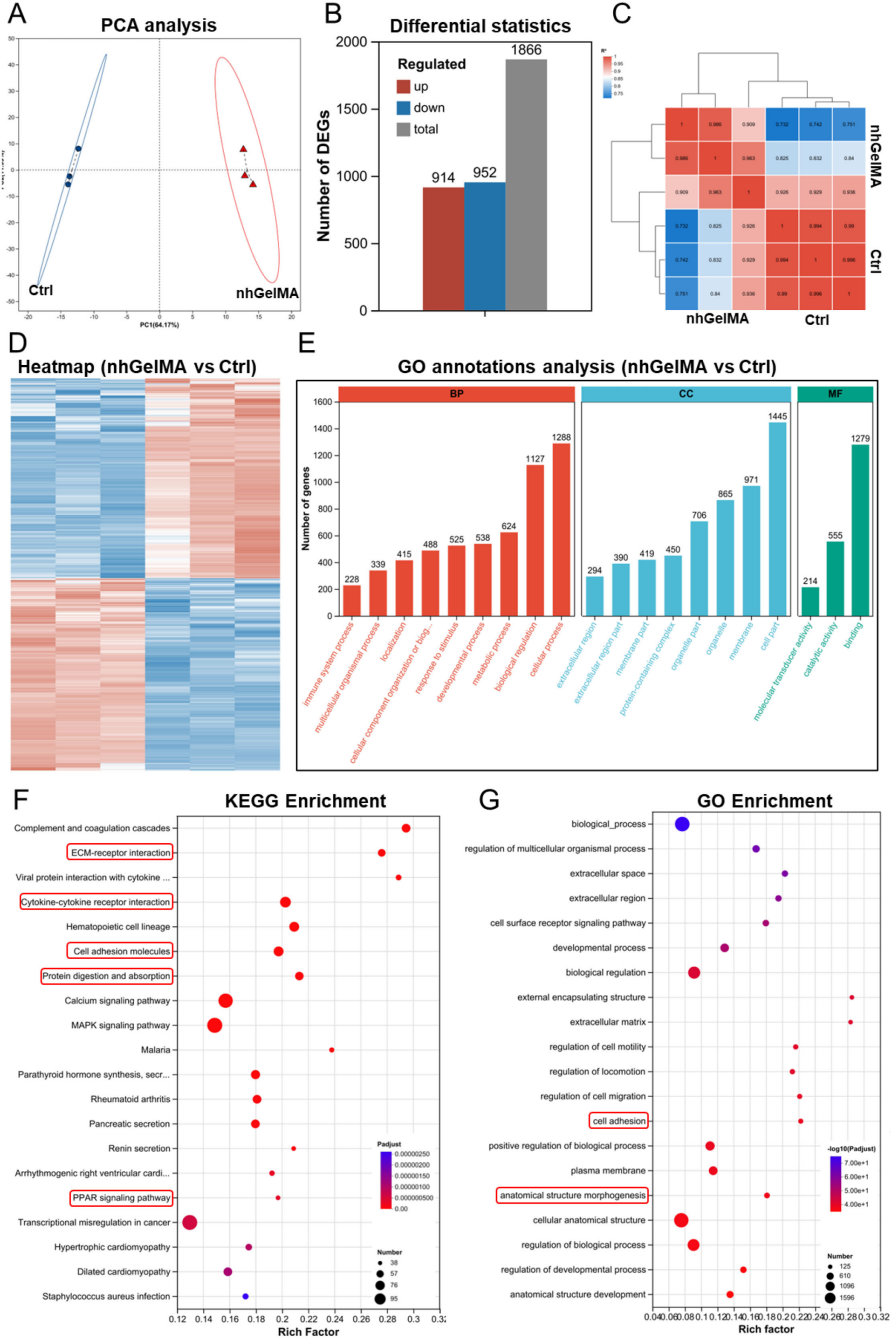
The mechanism analysis of nhGelMA in alleviating hemorrhagic intestinal adhesion. **(A)** PCA analysis of nhGelMA and Ctrl groups. **(B)** The differential expressive genes (DEGs) between nhGelMA and Ctrl groups. **(C)** Correlation analysis between nhGelMA and Ctrl groups. **(D)** Heatmap analysis in the two groups. **(E)** GO annotations analysis of DEGs in the two groups. **(F, G)** KEGG and GO enrichment analyses.

To further clarify the mechanism analysis of nhGelMA in alleviating hemorrhagic intestinal adhesion, transcriptomics was conducted on the nhGelMA and untreated (Ctrl) groups 3 days post-surgery. PCA and expression distribution analyses demonstrated good biological replicates within both the nhGelMA and Ctrl groups, suggesting the reliability of the experimental data (**Figure 8A**; **Supplementary Figure S1A**), which was further corroborated by correlation analysis (**Figure 8C**). The Venn diagram revealed that the nhGelMA group detected a total of 12,527 genes, of which 602 were unique. In contrast, the Ctrl group identified 12,386 genes, including 461 unique genes. Notably, both groups shared a total of 11,925 common genes (**Supplementary Figure S2A**). Differential statistics revealed that nhGelMA upregulated 914 genes and downregulated 952 genes, while 1866 genes exhibited no significant expression differences (**Figure 8B**). Heatmap and volcano plots of the differentially expressed genes indicated a number of significantly differentially expressed genes, suggesting that this strategy mitigates intestinal adhesion through various potential mechanisms (**Figure 8D**; **Supplementary Figure S2B**). Furthermore, GO functional annotation analysis showed that the differential genes were distributed across biological process, cellular component, and molecular function, indicating that the strategy for alleviating intestinal adhesion involves comprehensive regulation (**Figure 8E**). To elucidate the specific regulatory pathways associated with this strategy, KEGG and GO enrichment analyses were performed (**Figures 8F, G**). The results indicated that nhGelMA can regulate ECM-receptor interactions, as well as pathways Cytokine-cytokine receptor interaction, Cell adhesion molecules, Protein digestion and absorption, PPAR signaling pathway, cell adhesion, Cytokine-cytokine receptor interaction, and anatomical structure morphogenesis, to alleviate local adhesion while remodeling the local intestinal mucosal structure. Furthermore, the heatmap of Protein digestion and absorption, Cell adhesion molecules, PPAR signaling pathwayand Cytokine-cytokine receptor interaction (**Supplementary Figures S3A-D**) showed the upregulated and downregulated genes, which related to alleviation of hemorrhagic intestinal adhesion. Therefore, nhGelMA could regulate protein digestion, absorption, and adhesion, as well as reconstruct the anatomical structure, contributing to the alleviation of hemorrhagic intestinal adhesion.

## 3 Conclusion

Intestinal adhesion is one of the most prevalent clinical conditions, affecting millions of patients globally. However, effective strategies to decrease the incidence of intestinal adhesion remain insufficient. in this study, GelMA, Houttuynia cordata extract, and nintedanib were integrated to develop a dual-functional methacrylated gelatin (nhGelMA) that can regulate inflammation and antifibrotic functions. First, optimal concentrations of GelMA hydrogels were prepared and screened, followed by the selection of appropriate concentrations of Houttuynia cordata extract and nintedanib to prepare the final nhGelMA. Subsequently, nhGelMA was characterized, and its biocompatibility and biological functions were analyzed. The results indicated that nhGelMA exhibited good biocompatibility, no organ toxicity, and the ability to modulate the expression level of inflammation and FN. Finally, nhGelMA hydrogel was applied in the preventive treatment of hemorrhagic intestinal adhesion, and the results indicated that nhGelMA effectively reduced the deposition of FN, LAMA, and Col1, as well as decreased the infiltration of macrophages and neutrophils, thereby preventing the occurrence of intestinal adhesion. Importantly, the further transcriptomics demonstrated that nhGelMA could regulate protein digestion, absorption, and adhesion, as well as reconstruct the anatomical structure, contributing to the alleviation of hemorrhagic intestinal adhesion. In summary, we developed a novel dual-functions nhGelMA hydrogel for alleviating hemorrhagic intestinal adhesion via immunomodulation and anti-fibrosis, providing a promising strategy with clinical translational potential. Nevertheless, the current strategy still requires more support from intestinal adhesion models and more data from large animals before it can be applied in clinical settings.

## 4 Materials and methods

### 4.1 Preparation of nhGelMA

To prepare GelMA, 10 g gelatin (Aladdin, G108394) was added into 100 mL deionized water, and stirred in a water bath at 50 °C until fully dissolved. Subsequently, the solution was slowly added 6 mL methacrylic anhydride (Aladdin, M102519) was slowly added and the mixture was allowed to react in a 50 °C constant-temperature shaker for 6 hours. After the reaction, the solution was transferred into a dialysis bag with a molecular weight cutoff of 3.5 kDa and dialyzed in deionized water for 2 days. Following dialysis, the solution was centrifuged at 3000 rpm for 10 minutes, as well as the supernatant was collected, frozen at -80 °C, and then freeze-dried for 2 days to obtain GelMA precursor (29). GelMA hydrogel was prepared by mixing the 5% GelMA precursor and 0.2% lithium phenyl-2,4,6-trimethylbenzoylphosphinate (LAP) under 365 nm ultraviolet (UV) light excitation. Similarly, the nhGelMA hydrogel was prepared by mixing 5% GelMA, 0.2% LAP, Houttuynia cordata extract (10 mg/ml), and Nintedanib (20 ug/ml) with UV light excitation.

### 4.2 ¹H NMR analysis

To confirm the successful preparation of GelMA, nuclear magnetic resonance hydrogen spectroscopy (¹H NMR) was conducted on gelatin and GelMA. 10 mg gelatin and GelMA were dissolved in 0.5 mL deuterated dimethyl sulfoxide (DMSO, Sigma, 151831) containing 0.03% tetramethylsilane (TMS) as an internal standard. Subsequently, 0.4 mL of each solution was transferred into a 5 mm NMR tube, and ¹H NMR measurements were performed using a Bruker 600 MHz NMR spectrometer (Germany). After data collection, the spectra were processed using MestReNova software. Characteristic peaks were identified: GelMA exhibited additional peaks at approximately 5.3-5.8 ppm, corresponding to the vinyl protons from methacryloyl groups, thereby confirming the methacrylation process.

### 4.3 Mechanical property

The hydrogel samples were prepared with a 1 cm diameter and a 3 mm thickness. Compressive tests were conducted using a universal testing machine equipped with a 500 N loading (INSTRON 5982). Briefly, the samples were placed on the compression platen and compressed at a rate of 1 mm/min until they reached 80% of their original thickness. The compression modulus was obtained from the stress-strain curve, where the modulus is defined as the slope of this linear segment (30).

### 4.4 CCK-8 assay

To evaluate the cytotoxicity of hydrogels, a CCK-8 assay was conducted using fibroblasts. First, 20 μL of hydrogel was added to the well of a 96-well plate and allowed to solidify under 365 nm UV light excitation. Subsequently, fibroblasts were seeded at a density of 5×10³ cells per well with 100 μL culture medium. The plate was incubated at 37 °C in a incubator for 24 hours. Then, 10 μL CCK-8 reagent (DOJINDO, CK04) was added to each well with 90 μL culture medium, and the incubation continued for another 2 hours under the same conditions. The absorbance at 450 nm was measured using a microplate reader. Relative cell viability was calculated based on the blank group.

### 4.5 Rheological analyses and viscosity measurements

Rheological tests were conducted using a HAAKE MARS Rotational Rheometer equipped with parallel-plate geometry (P20 TiL, 20 mm diameter) at 25 °C. The rheology experiments involved exposing the samples to light irradiation (365 nm, 20 mW/cm²). A time sweep oscillatory test was conducted under the conditions of 10% strain, 1 Hz frequency, and 0.5 mm gap for 120 s, during which the storage modulus (G’) and loss modulus (G’’) were recorded. All tests were performed in triplicate, and the data were analyzed using the instrument’s software to generate relevant curves and viscoelastic parameters.

### 4.6 Swelling tests of the hydrogels

Swelling tests of the hydrogels were conducted using a gravimetric method. The initial wet weight of the hydrogel samples was recorded as W_0_. The hydrogels were subsequently immersed in PBS at 37 °C for 2 h, 4 h, 8 h, and 12 h. After this period, the hydrogels were carefully removed, and their wet weights were measured and recorded as W_t_. The swelling ratio at each time point was calculated using the formula: Swelling ratio = (W_t_ - W_0_)/W_0_ × 100%.

### 4.7 Degradation test of the hydrogels

Degradation tests of the hydrogels were performed by a gravimetric method. The initial dry weight of the lyophilized hydrogel samples was recorded as W_0_. The hydrogels were then immersed in PBS with 10µg/ml collagenase (Aladdin, C754929) at 37 °C. At designated time intervals 12 h, 24 h, 48 h, and 72h, the hydrogels were carefully removed, lyophilized, and their weights were measured and recorded as W_t_. The degradation ratio at each time point was calculated using the formula: Degradation ratio = (W_0_ - W_t_)/W_0_ × 100%.

### 4.8 Sustained released tests

Sustained release tests of houttuynia cordata extract from hydrogels were performed by an absorbance method. nhGelMA hydrogels containing 10 mg/ml houttuynia cordata extract were immersed in PBS at 37 °C with supernatants collected at 12 h, 24 h, 48 h, and 72 h. The standard curve was established by measuring the PBS solutions with 0 μg/ml, 1 μg/ml, 10 μg/ml, 100 μg/ml, 1000 μg/ml, 10 mg/ml, and 100 mg/ml houttuynia cordata extract. Then the standard curve plotted with concentration as the x-axis and OD value as the y-axis. The release rate of houttuynia cordata extract in the supernatants calculated based on the standard curve. Similarly. sustained release tests of Nintedanib from nhGelMA hydrogels were performed by a liquid chromatography method. nhGelMA hydrogels loaded with Nintedanib were immersed in PBS at 37 °C with supernatants collected at 12 h, 24 h, 48 h, and 72 h. The standard curve was harvested by measuring the release medium solutions with 0 ug/ml, 1 ug/ml, 10 ug/ml, and 100 ug/ml. Then the standard curve plotted with concentration as the abscissa and peak area as the ordinate. The release rate of Nintedanib in the supernatants calculated based on the standard curve.

### 4.9 SEM and element analysis

SEM observations and element analysis were performed using a ZEISS GeminiSEM 300 instrument (Germany). The lyophilized samples were sputter-coated with gold to enhance conductivity. SEM images were captured at accelerating voltage of 5 kV. Element distribution was analyzed via energy-dispersive spectroscopy (EDS) Mapping, which facilitated the detection and visualization of the elemental composition across the sample surface (31).

### 4.10 XRD and FTIR

XRD analysis was performed using a Rigaku SmartLab SE instrument (Japan) with a scanning angle range of 10-80° and a scanning speed of 2°/min. FTIR measurements were conducted using a Nicolet iS20 instrument (Thermo Fisher Scientific, USA) with a wavenumber range of 400–4000 cm^-1^ to characterize the functional groups of the samples.

### 4.11 Isolation and culture of fibroblasts

Rabbit skin tissue was harvested and rinsed with PBS containing antibiotics. The tissue was then cut into small pieces using sterile scissors. Subsequently, the pieces were immersed in PBS with 0.2% Dispase II (Aladdin, D743370) and incubated at 37 °C for 8 hours to facilitate the separation of the epidermis from the dermis. A 0.1% collagenase solution (Merck, C0773) was added, and the mixture was incubated at 37 °C for 2 hours to digest the dermal tissue. The cell suspension was filtered through a cell strainer to remove undigested tissue fragments, and the filtrate was centrifuged at 1000 rpm for 5 minutes to isolate the cells, which were subsequently cultured in DMEM supplemented with 10% fetal bovine serum and 1% antibiotics (32).

### 4.12 Live/dead staining

Cells were seeded onto the hydrogels and cultured for 72 hours. Following the removal of the culture medium, the samples were gently rinsed twice with PBS to remove residual medium. A Live/Dead staining solution (DOJINDO) was applied to cover the hydrogels, and the samples were incubated at 37 °C for 10 minutes. After incubation, the staining solution was aspirated, and the samples were then rinsed with PBS to remove any excess dye. The stained cells were then observed immediately under a fluorescence microscope (Leica), where live cells exhibited green fluorescence and dead cells emitted red fluorescence.

### 4.13 PCR

Total RNA was extracted from samples using a TRIzol reagent according to the manufacturer’s instructions. cDNA was synthesized from total RNA using a reverse transcription kit based on the kit protocol. PCR amplification was performed in a 20 μL reaction system, which included10 μL of 2× PCR Master Mix, 1 μL of forward primer (10 μM), 1 μL of reverse primer (10 μM), 2 μL of cDNA template, and 6 μL of nuclease-free water. The relative expression level of the target gene was calculated using the 2^(-ΔΔCt) method (33). Primers were designed and synthesized by Sangon Biotech (Shanghai).

### 4.14 Surgery and ethic

New Zealand white rabbits (2 kg, 2 months) were purchased from Shanghai Jiagan Biotechnology Co., Ltd. The rabbits were anesthetized using isoflurane, after which the abdominal area was depilated and disinfected. A midline abdominal incision was made to open the abdominal cavity, and the appendix was carefully identified and exposed. The appendix was scratched with a scalpel handle to create a bleeding model. In the experimental group, nhGelMA was applied to the injured area for treatment, while the control group received no treatment. After the procedure, the abdominal cavity was closed layer by layer. All animal procedures were approved by the Animal Care and Experimental Committee of Nantong University (S20250818-001).

### 4.15 Immunofluorescence staining

The rabbits were euthanized after 3 days repair and the intestines were harvested. The samples were fixed in 4% paraformaldehyde for 24 hours, followed by dehydration and sectioning at a thickness of 4-5 μm. The sections were dewaxed to water, subjected to antigen retrieval, and then blocked with 5% BSA at room temperature for 30 minutes. Primary antibodies against FN (Abcam, ab286324), LAMA (Abcam, ab151715), Col1 (Abcam, ab270994), CD3 (Abcam, ab16669), and CD68 (HUABIO, HA601292) were added to the sections, which were then incubated overnight at 4 °C for 12 h. After washing containing phosphate-buffered saline with Tween 20 (PBST), fluorescently labeled secondary antibodies were added, and the sections were incubated in the dark at 37 °C for 1 hour. Finally, the sections were counterstained with DAPI for 5 minutes and observed under a fluorescence microscope. Images were captured for subsequent analysis.

### 4.17 Statistical analysis

All data are presented as mean ± standard deviation (SD). A t-test was utilized for comparisons between two groups, whereas a one-way analysis of variance (ANOVA) was applied. A p-value less than 0.05 was considered statistically significant.

## Data Availability

The original contributions presented in the study are included in the article/**Supplementary Material**. Further inquiries can be directed to the corresponding author.
