# Supplementary material for: Alleviation of tissue adhesion using dual-functional methacrylated gelatin via immunomodulation and antifibrotic activity
Source: Front Immunol. 2026 Mar 4;17:1777773. doi: 10.3389/fimmu.2026.1777773 (PMC12995757; doi:10.3389/fimmu.2026.1777773)
Supplement: Supplementary file 1 [file DataSheet1.docx]

**Alleviation of tissue adhesion using dual-functional methacrylated gelatin via immunomodulation and antifibrotic activity**

*Pei Yuan*^1^, *Shichun Feng*^1^, *Chong Tang*^1^, *Xuesong Gao*^1^, *Shengkui Qiu*^1^, *Geshuyi Chen*^2^*

1. Gastrointestinal Surgery Department, Nantong First People's Hospital, Southeast University, Nantong, Jiangsu, China.

2. Gynecology Department, Nantong First People's Hospital, Southeast University, Nantong, Jiangsu, China.

corresponding author: *

Emial: [chengshy95@163.com](mailto:chengshy95@163.com)

**Key words:** biomaterials, hydrogel, tissue engineering, Houttuynia cordata extract, Nintedanib, intestinal adhesion

Figures


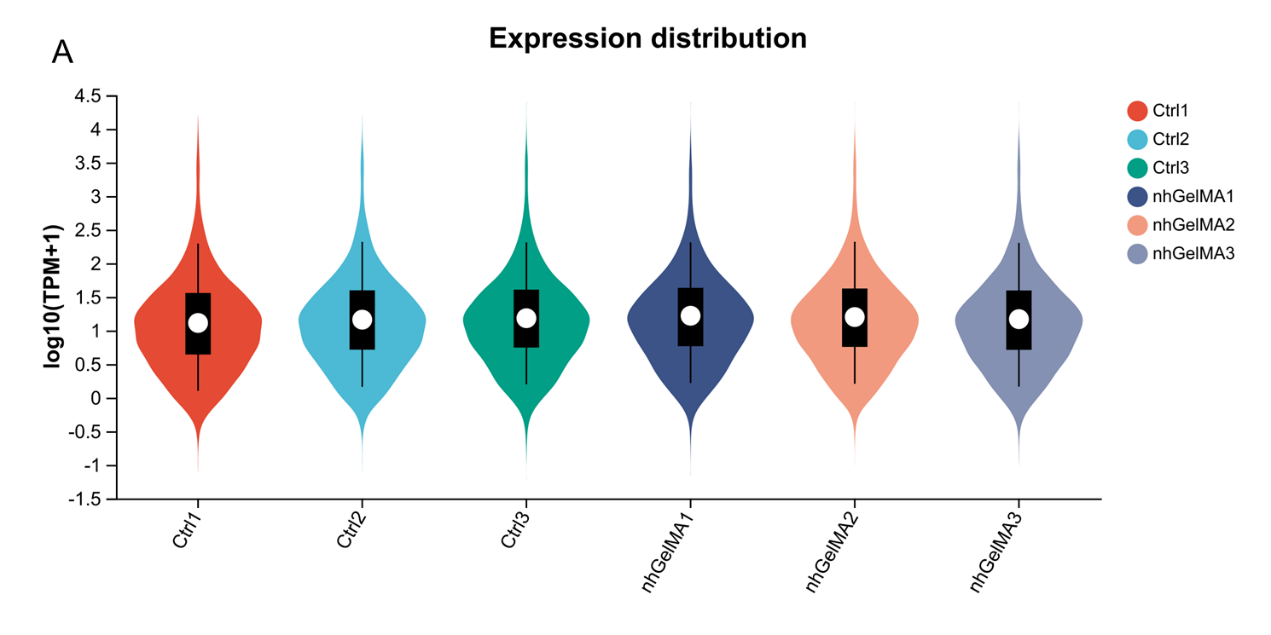


Figure S1. (A) The expression distribution between nhGelMA and Ctrl groups.


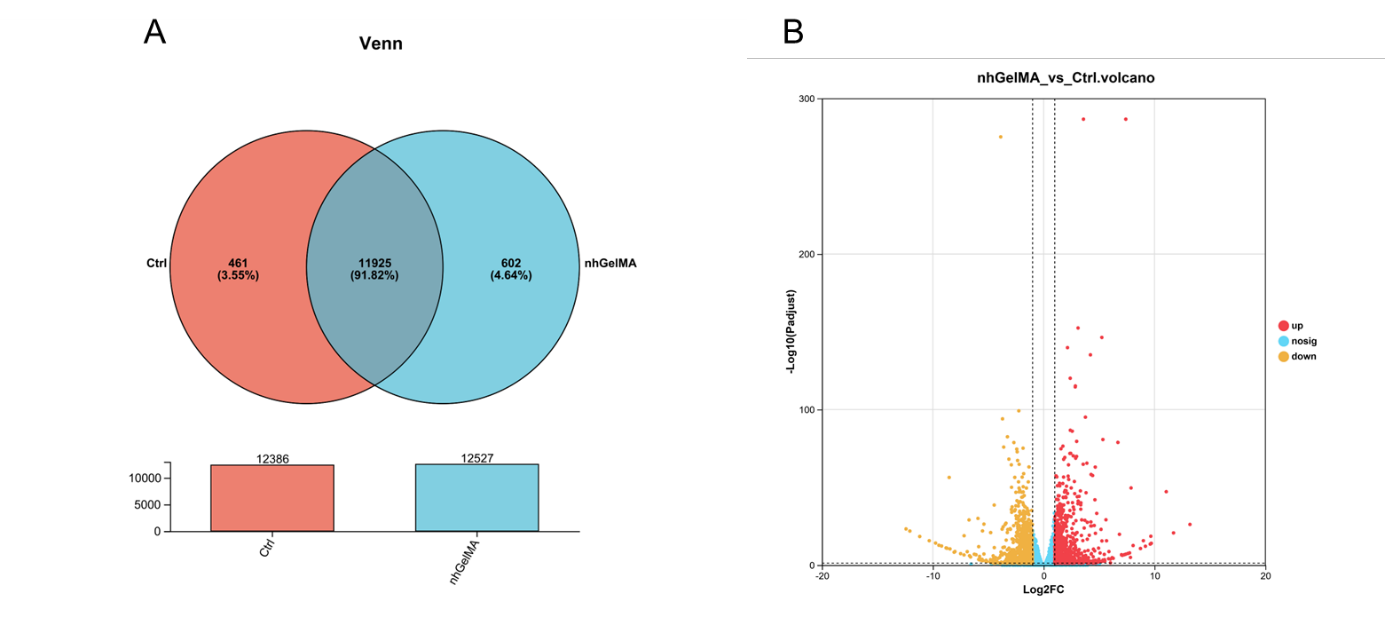


Figure S2. The venn (A) and volcano plots (B) between nhGelMA and Ctrl groups.


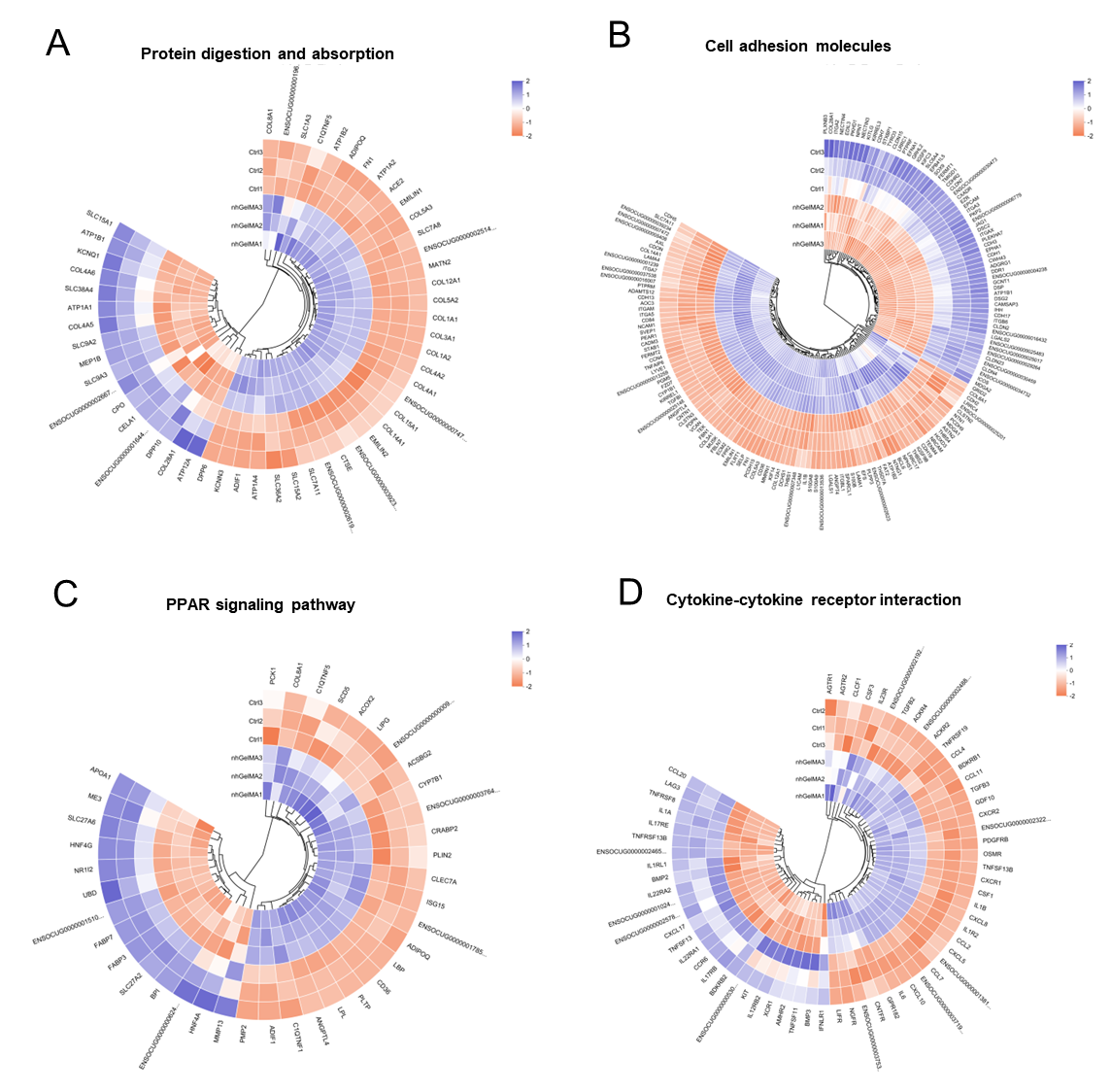


Figure 3. The heatmap of Protein digestion and absorption (A), Cell adhesion molecules (B), PPAR signaling pathway (C) and Cytokine-cytokine receptor interaction (D).
